# Identification of Sites of STAT3 Action in the Female Reproductive Tract through Conditional Gene Deletion

**DOI:** 10.1371/journal.pone.0101182

**Published:** 2014-07-01

**Authors:** Rebecca L. Robker, Laura N. Watson, Sarah A. Robertson, Kylie R. Dunning, Eileen A. McLaughlin, Darryl L. Russell

**Affiliations:** 1 Robinson Research Institute, School of Paediatrics and Reproductive Health, The University of Adelaide, Adelaide, South Australia, Australia; 2 School of Environmental & Life Sciences, University of Newcastle, Callaghan, New South Wales, Australia; Institute of Zoology, Chinese Academy of Sciences, China

## Abstract

The STAT3 transcription factor is a pleiotropic transducer of signalling by hormones, growth factors and cytokines that has been identified in the female reproductive tract from oocytes and granulosa cells of the ovary to uterine epithelial and stromal cells. In the present study we used transgenic models to investigate the importance of STAT3 for reproductive performance in these different tissues. The Cre-LoxP system was used to delete STAT3 in oocytes by crossing *Stat3^fl/fl^* with *Zp3-cre*+ mice, or in ovarian granulosa cells and uterine stroma by crossing with *Amhr2-Cre*+ mice. Surprisingly, deletion of STAT3 in oocytes had no effect on fertility indicating that the abundance of STAT3 protein in maturing oocytes and fertilized zygotes is not essential to these developmental stages. In *Stat3^fl/fl^*;*Amhr2-cre*+ females impaired fertility was observed through significantly fewer litters and smaller litter size. Ovulation rate, oocyte fertilization and development to blastocyst were unaffected in this line; however, poor recombination efficiency in granulosa cells had yielded no net change in STAT3 protein abundance. In contrast, uteri from these mice showed STAT3 protein depletion selectively from the stomal compartment. A significant reduction in number of viable fetuses on gestational day 18, increased fetal resorptions and disrupted placental morphology were evident causes of the reduced fertility. In conclusion, this study defines an important role for STAT3 in uterine stromal cells during embryo implantation and the development of a functional placenta.

## Introduction

Female reproductive organ function is controlled and coordinated by an intricate network of signalling hormones, growth factors and cytokines [1], [2]. The interaction of many of these signalling factors with their receptors can activate intracellular signal transducer and activator of transcription 3 (STAT3) [3]. STAT3 is a member of a family of cytoplasmic proteins which are phosphorylated after ligand interaction with cell surface receptors, then translocated to the nucleus and bind specific gene promoter elements to modulate expression. STAT3 protein is tyrosine phosphorylated by the Janus kinases (JAK) and by receptor tyrosine kinases in response to cytokine and growth factor signals.

In the ovary, oocytes sequestered in primordial follicles remain quiescent until recruited into the growing pool sporadically throughout the reproductive years. Follicle activation, growth and atresia are major biological checkpoints that control female reproductive potential and establish the developmental potential of the resultant embryo [4]. Hormones, growth factors and cytokines all contribute to the control of follicle activation and development [2], [5]. High levels of STAT3 and activated phospho-STAT3 have been detected in the granulosa cells, oocytes and theca cells [6], [7] of growing follicles. Abundant STAT3 has been demonstrated in oocytes at all stages of follicular development, although its cytoplasmic localisation has suggested the majority is usually not transcriptionally active in oocytes [6], [8]. However cytokines that signal via STAT3 are thought to act on oocytes [9], suggesting that STAT3 may be a key mediator of regulated gene transcription in the oocyte.

Important signalling factors known to act on ovarian granulosa cells and also known to signal through STAT3 activation include epidermal growth factor (EGF; [10]), leukemia inhibitory factor (LIF; [11]), interleukin 6 (IL6; [6]) and leptin [12]. In porcine granulosa cells STAT3 can be phosphorylated in response to EGF treatment [7] or leptin [13] in vitro. Active DNA-binding STAT3 was identified in the granulosa cells of immature follicles from hypophysectomised rats [14], [15]. We have also found induction of the Stat3 responsive gene and functional regulator of STAT, SOCS4, in early activated primary follicles [11]. These findings led to our hypothesis that STAT3 and SOCS4 are regulators that mediate follicle activation and the transition of primordial into primary follicles. However, very little is known about the actions of STAT3 in granulosa cell or oocyte function and how STAT3 influences follicle activation or growth/atresia.

Similarly, in the preimplantation embryo, oocyte-derived STAT3 is implicated in the first cleavage cycles in regulating responsiveness to endogenous and maternal tract-derived cytokines and growth factors including EGF, HB-EGF, LIF and IL-6 [16], [17]. STAT3 is essential for normal embryogenesis, and STAT3 null mouse embryos die shortly after implantation.

In the uterus, STAT3 regulating growth factors such as EGF [10] and cytokines including LIF, IL-6 and leptin, released by epithelial cells and infiltrating immune cells play key roles regulating the differentiation during decidualisation and the implantation process. In the uterus, LIF acts on the luminal epithelium and trophoblast to promote embryo attachment, while IL11 acts on the epithelial and endometrial stromal cells stimulating decidualisation and invasion of the extravillous trophoblast [18]. IL-6 is involved in several pathways and processes that impact on placental development and function [19]. STAT3 is a key mediator of the response to each of these cytokines, as well as EGF and other growth factors and is known to contribute to preparing the endometrium for implantation. Inhibition of activated STAT3 function has been shown to block implantation when applied locally to the uterus [20]. Recently STAT3 has been shown to be necessary for mediating normal receptivity to embryo attachment in uterine epithelial cells [21] where it interacts with Progesterone Receptor [22]. Deletion of STAT3 in the progesterone receptor (Pgr) expressing uterine cells (epithelium and stroma) reduced the expression of PR and completely prevented implantation [22]. However, the selective role for STAT3 in stromal differentiation and preparation for implantation is unknown.

Here we used several mouse genetic models intended to conditionally delete STAT3 selectively in oocytes, ovarian granulosa cells and in uterine stromal cells to investigate the roles of this important signalling mediator in folliculogenesis, ovulation and implantation. Using *ZP3-Cre* strain mice which selectively express Cre recombinase in oocytes from late primaryfollicle stage we demonstrate that although it is abundant in oocytes, efficient ablation of STAT3 in *Stat3^fl/fl^*;*Zp3-Cre* ^+^ females did not affect development of competent fertilizable oocytes. The Amhr2 promoter drives Cre expression from embryonic day e12.5 in the Müllerian duct which derives the adult oviducts, uterine stroma and myometrium as well as from postnatal day 8 in granulosa cells from the secondary follicle stage. Interestingly, using the AmhR2-Cre transgenic mouse line in conjunction with STAT3^fl/fl^ mice did not achieve efficient protein depletion in granulosa cells. However, STAT3 ablation in uterine stromal fibroblasts in *Stat3^fl/fl^*;*Amhr2-Cre* ^+^ mice demonstrated a key role for STAT3 in these cells for implantation, placental development and also potentially for postpartum remodelling of the uterus.

## Materials and Methods

### Materials

Equine chorionic gonadotropin (eCG) was purchased from Intervet Pty Ltd, Australia. Human chorionic gonadotropin (hCG) was purchased from Calbiochem, Australia (Alexandrina, NSW). Culture media was purchased from GIBCO, Invitrogen Australia Pty. Ltd. Antibodies were purchased from Santa Cruz Biotechnology Inc (goat anti-rabbit IgG-HRP (sc-2004), Cell Signaling Technology (anti-STAT3, 9132), Chemicon, Boronia, VIC (biotinylated goat anti rabbit), Sigma (anti-β-actin, A3854), and Abcam (goat pAb to mouse IgG (HRP) ab6789-1)). Unless stated, all other reagents were purchased from Sigma Aldrich Pty Ltd (NSW, Australia).

### Generation of conditional knockout mice and genotyping strategy

All mice were maintained on a 12∶12 h day/night cycle with rodent chow and water provided *ad libitum*. All murine experiments were approved by the University of Adelaide's Animal Ethics Committee (approval #M-081-2007) and were conducted in accordance with the Australian Code of Practice for the Care and Use of Animals for Scientific Purposes (2013). Euthanasia was by cervical dislocation by a trained, experienced and accredited technician.

Floxed *Stat3* mice with exons 11 to 14 flanked by two loxP sites were generated by Alzoni et al. 2001 [23]. Mice were genotyped for the floxed *Stat3* allele (*STAT3^fl/lf^* or *STAT3^fl/+^*) by performing PCR using the following primers: Flox11F 5′-CACCAACACATGCTATTTGTAGG-3′ and Flox11R 5′-CCTGTCTCTGACAGGCCATC-3′. In order to generate animals in which the *Stat3* gene was non-functional, *Stat3* floxed mice were crossed with one of two Cre transgenic lines, *Amhr2-Cre* or *ZP3-Cre*. *Amhr2-Cre* knock in mice [24] were genotyped for the presence of the Cre allele by PCR using the following primers: BPA-F 5′- CGCATTGTCTGAGTAGGTGT-3′ and MISR-R 5′-GAAACGCAGCTCGGCCAGC-3′. *ZP3-Cre* knock in mice (The Jackson Laboratory, stock #003651) were genotyped for the presence of the Cre allele by PCR using the following primers: IMR1084 5′-GCGGTCTGGCAGTAAAAACTATC-3′ and IMR1085 5′-GTGAAACAGCATTGCTGTCACTT-3′.

### Confirmation of Recombination by PCR

To asses recombination in target tissues (GC and oocytes), DNA was extracted and PCR was performed using the following primers: Flox11F (as described above) and Flox14R 5′-GCAGCAGAATACTCTACAGCTC-3′. The Wild type *Stat3* allele produces a 2100 bp amplification product with these primers while, if recombination had occurred, a 310 bp product was observed in 2% agarose gel electrophoresis stained with Gel Red (BioRad).

### Isolation of COCs and GC

Mice were humanely euthanized at times specified in results and COCs and GCs were collected in minimum essential medium (MEM) (alpha MEM from Gibco, Invitrogen) by puncture of follicles (or puncture of the ampulla of the oviduct for ovulated COCs) with a 26 gauge needle to release cells. When super-ovulation was required, mice aged 21-25 days were administered 5IU eCG by i.p. injection, followed 44 hours later by 5 IU of hCG. Cells were collected and pelleted, and excess media removed before being snap frozen in liquid nitrogen and stored at −80°C until use.

### Western Blot

For the *Stat3*x*Amhr2-Cre* strain, the level of total STAT3 protein present in GC was assessed by Western blot. Cell pellets were resuspended in 0.05 M sodium acetate, 6 M urea and 0.1% Triton buffer and protease inhibitors (α protease inhibitor cocktail (10 µl/ml) and Aprotinin (10 IU/ml)). Homogenisation was carried out by vortexing followed by gentle agitation for 1 h at 4°C. The extract was then centrifuged at 10,000x*g* for 5 min and the supernatant transferred to a fresh tube. Protein concentrations were determined by Bradford assay (Bio-Rad Laboratories Pty. Ltd.). Proteins were separated on a reducing 10% acrylamide gel then transferred to a polyvinylidene difluoride membrane (Immobilon-P, Millipore Corporation). Membranes were blocked in TBST (10 mM Tris-HCl pH 7.5, 150 mM NaCl and 0.05% Tween 20) containing 3% non-fat milk for 1 at room temperature. Membranes were then incubated with primary antibodies in 3% milk/TBST for 2 h at room temperature (STAT3, 1∶1000 and β-actin, 1∶10,000). Blots were then washed in TBST and incubated with secondary antibodies, (goat anti-rabbit IgG-HRP, 1∶2000 or goat anti-mouse IgG-HRP, 1∶5,000) for 1 h at room temperature, and then blots were again washed in TBST. Enhanced chemiluminescence detection was performed as per manufacturer's instructions (Amersham, GE Healthcare Life Sciences, Ryldamere NSW, Australia).

### Fertility assessment

To asses reproductive performance, 6–8 week old female wild type (*Stat3^fl/fl^;Cre^-^*), STAT3 heterozygous (*Stat3^fl/+^;Cre^+^*) and STAT3 knock out (*Stat3^fl/fl^;Cre^+^*) mice were mated with proven C57Bl6 male mice, and pup numbers recorded over a 26 (*Stat3-ZPCre*) or 30 (*Stat3-AmhrCre*) week period. Stud males were switched between cages after 3 months.

### Histological assessment of reproductive organs/Immunohistochemistry

Tissues were fixed in 4% paraformaldehyde, embedded in paraffin and then cut into 7 µm sections before being mounted on glass slides. Tissue sections were dewaxed and rehydrated and antigen retrieval was performed by incubating slides for 10 min at RT in proteinase K (10 µg/ml). Sections were rinsed in PBS before being treated with 3% hydrogen peroxide for 10 min. Sections were washed sequentially in PBS then PBST (PBS with 0.025% Tween-20) before being blocked with 10% normal goat serum (NGS) in PBST for 1 h in a humid chamber. Primary antibody (anti-STAT3, 1∶1000) was applied and incubated overnight in a humid chamber at room temperature. Negative control slides had the primary antibody omitted. Sections were washed thoroughly in PBST before incubation with the secondary antibody, biotinylated goat anti rabbit (1∶500) for 1 h in a humid chamber at room temperature. Protein localisation was visualised with the VECTASTAIN ABC Kit and the Vector DAB Substrate Kit (both Vector Laboratories Inc.), according to the manufacturer's directions. Sections were briefly counterstained with diluted haematoxylin then dehydrated and air dried before mounting in Permount (Fisher Scientific). Images were captured on a NonoZoomer digital imager and intensity of immunostaining was analysed using phase analysis with AnalySIS software (Olympus). To verify reduced STAT3 protein in oocytes, AnalySIS-ProTM software (Soft Imaging System, Germany) was used. Briefly, a colour threshold for positive immunostain was set based on the intensity of positive staining (brown pixels) in granulosa cells of wild type mice. This threshold was then assessed in defined regions of equivalent area in 20 randomly selected oocytes from each of the three genotypes and results were expressed as the percentage of pixels that were positive (at or above the threshold brown intensity) per field. No background subtraction was applied. Analysis was based on the average results from 20 oocytes in one representative section from each (n = 4–5) animal of each genotype.

### Natural ovulation rate and embryo development analysis of the *STAT3*x*Amhr2-Cre* strain

Female *Stat3^fl/fl^;Amhr2-Cre* and *Stat3^fl/+^*; *Amhr2-Cre* at 6-8 weeks old were paired with proven C57Bl6 males. Each morning females positive for a vaginal mating plug were humanely euthanized and presumptive zygotes flushed from the oviduct and counted. Zygotes were cultured in G1 (Vitrolife) fertilization media and then moved to fresh G2 embryo culture media at the 2-cell stage and cultured to the blastocyst stage. Each day the numbers of embryos at each developmental stage were scored.

### Statistical analysis

Data were analysed for statistical significance using Graph Pad Prism software version 5.01 (City CA, USA), using tests as outlined in the results for each experiment. A *P*-value <0.05 was considered statistically significant.

## Results

### Mice with oocyte-specific deletion of STAT3 (*Stat3^fl/fl^*;*Zp3-cre* ^+^) have no infertility phenotype

Phosphorylated active STAT3 has been reported in oocytes in a number of studies [6], [8] and here we confirmed abundant STAT3 in oocytes of follicles at stages from early primary to preovulatory. To assess the physiological importance of oocyte STAT3 we crossed *Zp3cre^+^* and *Stat3^fl/fl^* mice, producing *Stat3^fl/fl^;Zp3-cre^+^* females with selective ablation of STAT3 in oocytes.

**Figure 1:**
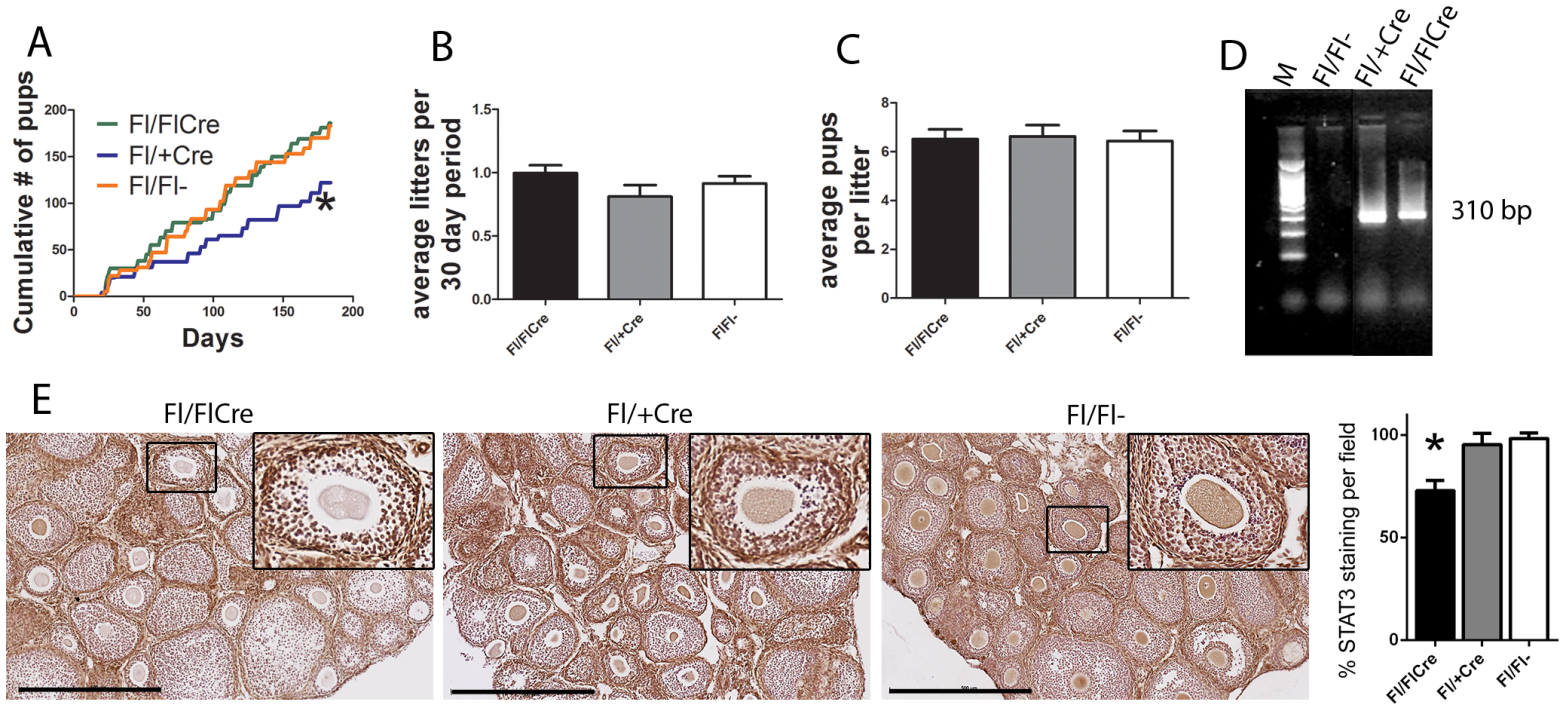
Normal fertility in oocyte STAT3 mutant *Stat3^fl/fl^;Zp3-cre^+^*mice. Reproductive ability of the *Stat3;ZP3Cre* line was tracked over a 26 week breeding period. **(A)** Cumulative number of pups (asterix indicates statistical significance from other groups, *P* < 0.05, one way ANOVA and Tukey's *post hoc* test). (**B**) average number of litters per 30 day period. (**C**) average number of pups per litter. (n = 5 breeder pairs per genotype). (**D**) Recombination of the *Stat3* gene in COCs was confirmed by PCR with the amplification of a 310 bp truncated product in heterozygous (*Stat3^fl/+^; Zp3-cre^+^*) and knock out (*Stat3^fl/fl^;Zp3-cre^+^*) mice. (**E**) STAT3 immuno staining (brown) in ovaries from mice at 26 days old, bar  = 500 µm.

Fertility was compared in the oocyte mutant *Stat3^fl/fl^; Zp3-cre^+^* females and control genotypes by evaluating production of offspring in breeding pairs over a 26 week period. Female control mice *(Stat3^fl/fl^;Zp3-cre^-^*, *Stat3^fl/+^;Zp3-cre^+^*) or oocyte null (*Stat3^fl/fl^;Zp3-cre^+^*) were mated with proven wild type C57Bl/6 stud males. A small reduction in cumulative pups born in the heterozygous (*Stat3^fl/+^;Zp3-cre^+^*) group was significant compared to both the Cre-negative and mutant (*Stat3^fl/fl^;Zp3-cre^+^*) groups (FIG 1A). However, this was interpreted as a statistical anomaly since the number of litters born each 30 day period over 26 weeks (FIG 1B) and number of pups born per litter (FIG 1C) were both not significantly different between all three genotype groups.

To confirm deletion of the *Stat3* gene in oocytes, PCR of DNA extracted from COCs collected from mice which had been super-ovulated was undertaken. The PCR conditions used did not amplify the 2100 bp product of the non-recombined *Stat3* allele in *Stat3^fl/fl^;Zp3-cre^-^* mice, while the predicted 310 bp band produced from the truncated gene after recombination was detected in heterozygous (*Stat3^fl/+^; Zp3-cre^+^*) and knock out (*Stat3^fl/fl^;Zp3-cre^+^*) oocytes showing that ZP3-Cre driven recombination occurred in these genotypes (FIG 1D). Immunostaining for STAT3 protein was consistently reduced in *Stat3^fl/fl^;Zp3-cre^+^* oocytes, further demonstrating effective gene deletion (FIG 1E). There were no obvious morphological differences between ovaries of each genotype, and positive staining in somatic cells remained consistent between the three genotypes. Negative controls showed in no visible immunostaining (data not shown).

This result shows that *Stat3* gene disruption in oocytes, and thus cleavage stage embryos, did not alter oocyte development or their capacity to generate live offspring at normal frequency.

### *STAT3^fl/fl^*;*Amhr-Cre* ^+^ mice are sub fertile

**Figure 2:**
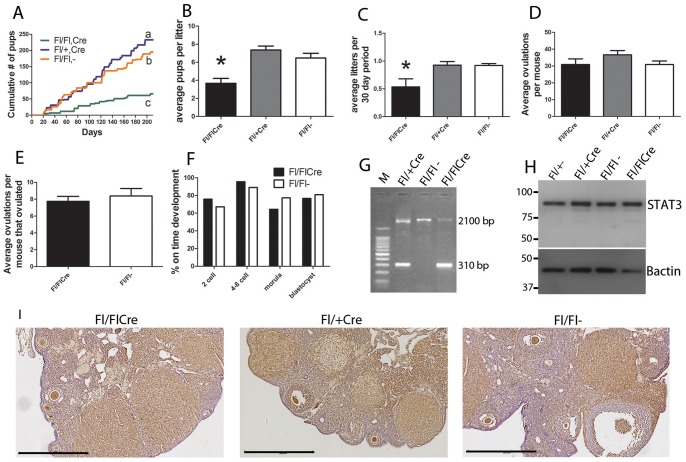
Subfertility in *Stat3^fl/fl^;Amhr2-cre^+^* mice. Reproductive ability of the *Stat3;Amhr2Cre* line was tracked over a 30 week breeding period. (**A**) Cumulative number of pups born (characters indicate statistical significance, *P*<0.05, one way ANOVA and Tukey's *post hoc* test). (**B**) Average number of litters per 30 day period. (**C**) Average number of pups per litter (asterix indicates statistical significance from other groups,*P*<0.05, one way ANOVA and Tukey's *post hoc* test) (n = 5 breeder pairs per genotype). (**D**) Ovulation rates were assessed in both hormonally stimulated (eCG+hCG) and naturally mated (**E**) mice (n≥8 mice per group). (**F**) Rate of embryo development was assessed in knock out and wild type groups following in vitro fertilization (n≥6 mice per group). (**G**) Recombination of the *Stat3* gene in COCs was confirmed by PCR with the amplification of a 310 bp truncated product in heterozygous (*Stat3^fl/+^;Amhr2-cre^+^*) and knock out (*Stat3^fl/fl^;Amhr2-cre^+^*) mice. (**H**) Abundance of STAT3 protein was analysed by Western blot of GC collected from all 4 genotypes of the *STAT3;Amhr2Cre* line. Pooled GC samples from 3 mice shown, equivalent sample loading (10 µg protein extract per lane) was confirmed by subsequent β-actin analysis of the same blot. (**I**) STAT3 immuno staining (brown) in ovaries from mice after completion of the 30 week breeding period, bar  = 500 µm.

*Stat3* was selectively mutated in granulosa cells of the ovary by crossing *Amhr2-cre* and *Stat3^fl/fl^* mice, producing *Stat3^fl/fl^;Amhr2-cre* females. Fertility was assessed in *Stat3^fl/fl^;Amhr2-cre^+^* female mice compared with controls (*Stat3^fl/fl^;Amhr2-cre^-^*, and *Stat3^fl/+^;Amhr2-cre^+^*) by pairing with proven C57Bl/6 stud males and evaluating litter production over a 30 week period. The cumulative number of pups per litter and number of litters was recorded. Importantly one of five *Stat3^fl/fl^;Amhr2-cre^+^* females was totally infertile, producing no offspring over the 30 week breeding period, though the fertility of the male was proven with other females. Considering only the remaining four breeders, there was a significant reduction in total pups born to the null (*Stat3^fl/fl^;Amhr2-cre^+^)* females compared to the *Stat3^fl/+^* heterozygous and Cre-negative control breeders over this period (FIG 2A). The heterozygous *Stat3^fl/+^;Amhr2-cre^+^* breeders had significantly increased cumulative pups born compared to the Cre-negative group. The average number of pups born per litter in the granulosa mutant group was reduced (by 44% compared to the Cre-negative controls 3.67 vs 6.47 pups/litter; FIG 2B, P = <0.0001). The average number of litters per 30 day period was also substantially lower, with 42% fewer litters in the null group compared to the controls (0.54 vs 0.92 litters/30 day period; FIG 2C, P = 0.0171).

The numbers of oocytes ovulated following eCG+hCG stimulated superovulation was not significantly affected by genotype (FIG 2D), demonstrating the absence of any adverse effect of genotype on generation of mature oocytes or the ovulation event when exogenous gonadotrophins are provided. To ensure an ovulatory defect was not masked by hormonal hyperstimulation we also assessed ovulation the morning after mating in natural estrus cycling adult females. Notably, although no significant effect was recoded, incomplete responsiveness in the GC null group was seen with 27% of mice (3 out of 11) failing to ovulate, while all mice from the control group ovulated successfully. This may reflect an ovarian defect in a proportion of mice, consistent with the one in five infertile females. Amongst the remaining naturally ovulating mice, no significant difference was observed in the number of cumulus oocyte complexes released (FIG 2E).

To determine whether fertilization or embryo development was affected after granulosa cell specific *Stat3* mutation, zygotes were cultured and the progression of embryo development was examined. The rates of fertilization (2-cell embryos after 24 h) and on-time embryo development to blastocyst were each equivalent in the null and Cre-negative controls (FIG 2F). Together, these results imply that the smaller litter size and less frequent litter production seen in *Stat3^fl/fl^;Amhr2-cre^+^* mice is the consequence of implantation success, or post-implantation developmental competence.

To evaluate recombination excision of the *Stat3* gene in granulosa cells, PCR was conducted on DNA extracted from COCs collected after superovulation of mice aged 23–25 days. The 310bp truncated gene product was detected in all *Stat3^fl/fl^;Amhr2-cre^+^;*and *Stat3^fl/+^* mice tested, but unexpectedly, wild type alleles of 2100 bp were also always detected (FIG 2G). Western blot showed no overt difference in STAT3 protein abundance in extracts of GC collected from non-stimulated mice 23–28 days old of all 4 possible genotypes of the Stat3;Amhr2cre line (*Stat3^fl/fl^;Amhr2-cre^-^*, *Stat3^fl/+^;Amhr2-cre^+^, Stat3^fl/fl^;Amhr2-cre^+^*, and *Stat3^fl/+^;Amhr2-cre^-^*)(FIG 2H). Absence of detectable change in STAT3 abundance in ovaries was further supported by STAT3 immunohistochemistry. Sections of ovaries from all mice at the completion of a 30 week breeding period were immunostained for STAT3. Negative controls showed no visible immunostaining (data not shown). There were no obvious morphological differences in ovarian structure observed, and positive staining for STAT3 in granulosa and luteal cells did not differ in localisation or abundance between the three groups (FIG 2I).

### Abnormal uterine structure and reduced fetal viability in *Stat3^fl/fl^*; *Amhr2-cre* ^+^ mice

**Figure 3:**
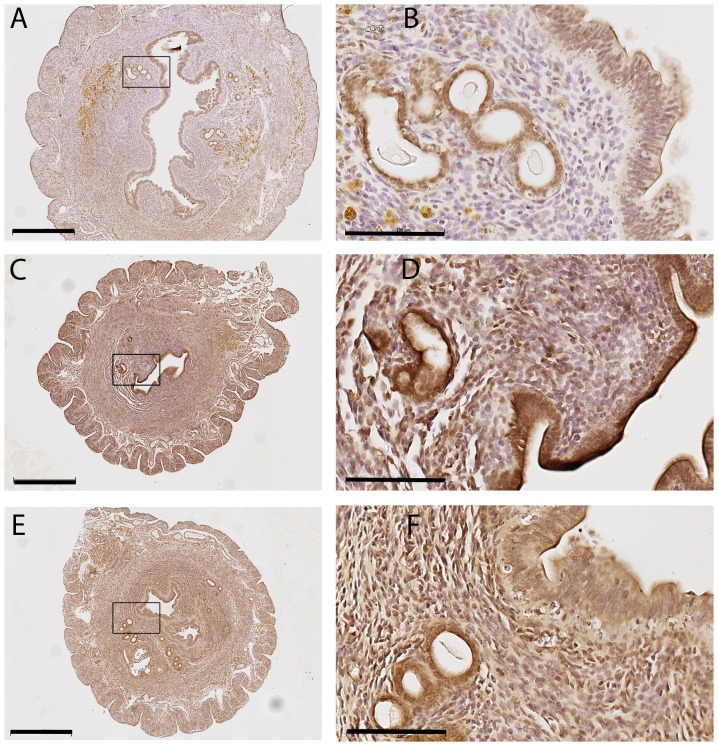
STAT3 deficiency in uterine stroma and aberrant uterine morphology in *Stat3^fl/fl^; Amhr2-cre^+^* mice. Transverse sections of uteri immunostained for STAT3 demonstrate the lack of STAT3 in **(A, B)** *Stat3^fl/fl^;Amhr2-cre^+^* uterine stromal cells, while luminal and glandular epithelial cells had equivalent STAT3 in all genotypes. **(C, D)** *Stat3^fl/fl^;Amhr2-cre^-^*, **(E, F)** *Stat3^fl/+^;Amhr2-cre^+^.* Representative examples of 5 mice for each genotype are shown. Scale bars in A, C and E 500 µm, in B, D, and E 100 µm.

The Amhr2 promoter drives Cre expression in cells of the Müllerian duct which are the progenitors of the oviducts, uterine stroma and myometrium in females from embryonic day e12.5 [24]–[26]. In order to investigate further the cause of the sub-fertility of the *Stat3^fl/fl^;Amhr2-cre^+^* mice, considering that no effect on oocyte development and no reduction in STAT3 within granulosa cells could be demonstrated, STAT3 abundance was investigated in the uterus. Immunohistochemistry in sections of uterine tissue collected from females at the completion of a 30 week breeding period revealed a clear reduction in STAT3 staining in the uterine stroma of *Stat3^fl/fl^; Amhr2-cre^+^* (FIG 3A,B) compared to controls (FIG 3C-F), while positive staining in luminal and glandular epithelial cells was consistent between the three genotypes (FIG 3). Negative controls showed no visible immunostaining (data not shown).

**Figure 4:**
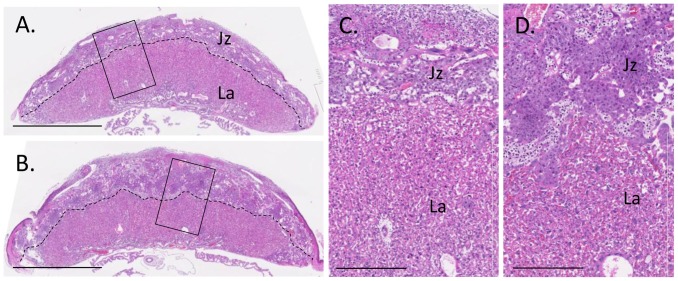
Reduced fetal viability and placental morphology in *Stat3^fl/fl^;Amhr2-cre^+^* mice. Mid-saggital sections of placental tissue recovered on day 17.5 pc from *Stat3^fl/fl^; Amhr2-cre^+^* females **(A, C)** and *Stat3^fl/+^;Amhr2-cre^+^* females **(B, D)** stained with hematoxylin and eosin. A moderately thicker junctional zone (Jz) compared with control genotypes was observed in *Stat3^fl/fl^;Amhr2-cre^+^* mice, while the labyrinthine (La) zone appears comparable in thickness. Representative images are shown. Representative examples of 7-9 mice per genotype. Bars in A&B are 2 mm; bars in C and d are 400 µm

To determine whether implantation and gestation were affected, females of each genotype were mated with wild type males. Again, fewer *Stat3^fl/fl^; Amhr2-cre^+^* females with evidence of progression from mating to pregnancy was seen, although this did not reach statistical significance. Of those that were pregnant at day 18 after observation of a mating plug, the fetuses, resorption sites and placentae were examined at autopsy. Although there was no difference in the total number of implantation sites, significantly fewer viable fetuses and more resorption sites were seen in *Stat3^fl/fl^;Amhr2-cre^+^* females, where the number of viable implants was substantially reduced by 39-49% compared to all other control groups (Table 1, P<0.05) and the number of resorption sites was increased at least 6-fold (Table 1). In viable implantation sites, the weight of viable fetuses was similar across genotypes but placental weight was substantially increased by approximately 27% in *Stat3^fl/fl^;Amhr2-cre^+^* mice and hence the fetal/placental weight ratio, a measure of placental functional competence, was reduced by approximately 22%. Consistent with this, histological assessment of placentas from *Stat3^fl/fl^;Amhr2-cre^+^* females revealed a notably thickened junctional zone compared to that of controls (FIG 4). This increase in fetal loss and abnormal development in *Stat3^fl/fl^;Amhr2-cre^+^* mothers is consistent with the poor performance of the breeding colony and observations of no change in ovulation or preimplantation embryo development.

**Table 1:** The effect of maternal STAT3 deficiency on fetal and placental parameters at day 18 of pregnancy.

	*Stat3^fl/+^;Amhr2-cre^-^*	*Stat3^fl/+^;Amhr2-cre^+^*	*Stat3^fl/fl^;Amhr2-cre^-^*	*Stat3^fl/fl^;Amhr2-cre^+^*
Females pregnant/females mated (%)^ a^	8/9 (89)	7/8 (88)	9/10 (90)	5/7 (71)
Mating interval (days)	6.2±1.6	2.9±0.9	3.2±0.9	2.5±0.7
Implantation sites/litter	7.6±0.4	8.4±0.3	8.4±0.5	9.0±0.9
Viable fetuses/litter	6.9±0.5 (90) ^a^	7.7±0.4 (92) ^a^	8.2±0.5 (97) ^a^	4.2±0.7 (46) ^b^
Resorbed fetuses/litter	0.8±0.4 (10) ^a^	0.7±0.3 (8) ^a^	0.2±0.1 (3) ^a^	4.8±0.4 (54) ^b^
Fetal weight (mg)	936±28	905±23	869±20	900±49
Placental weight (mg)	110±3 ^a^	103±2 ^a^	108±2 ^a^	135±5 ^b^
Fetal: placental weight ratio	8.7±0.4 ^a^	8.8±0.2 ^a^	8.1±0.2 ^ab^	6.9±0.5 ^b^

Values are mean ± SD. Data were compared by independent samples t-test. Values with different superscript characters were significantly different P<0.05.

## Discussion

STAT3 is expressed in multiple female reproductive tissues where it potentially transduces signals from many cytokines and growth factors that influence reproductive processes. We previously found that the *Stat3* target gene and modulator of STAT action SOCS4 is induced in early primordial follicle activation [11]. We undertook conditional deletion of STAT3 *Stat3* in oocytes, granulosa cells and uterine stromal cells in order to better determine the key sites of STAT3 action in female fertility. The results provide evidence that maternal STAT3 plays a critical role specifically in the uterine stroma, and is necessary for normal establishment of the feto-maternal interface and placental development. Collectively the data reported herein show that STAT3-deficiency in AMHR-expressing tissues results in a 50% reduction in fecundity, fully accounting for loss occurring in the post-implantation phase of pregnancy.

STAT3 is strongly expressed in uterine luminal and glandular epithelium [21], [27] as well as stromal cells [28] and is also known to be phosphorylated and activated in epithelial and stromal cells during the window of implantation receptivity, around 3.5 and 5.5 dpc respectively [28]-[30]. A previous report of *Stat3* gene ablation simultaneously in epithelial and stromal cells of the uterus by crossing *Stat^fl/fl^* mice with progesterone receptor-promoter driven Cre expressing mice caused implantation failure [22]. These mice exhibited dysregulated expression of *Pgr* and other luminal epithelial genes involved in embryo attachment and invasion, clearly defining a role for STAT3 in conjunction with *Pgr* in the response to embryo-endometrial interaction. However, the importance, if any of STAT3 in uterine stroma was masked by the severe defect in epithelial function and embryo implantation and hence was not identified in that study. In the current study, we have deleted uterine STAT3 selectively in the myometrial and stromal cells of uteri where *Amhr2* is strongly expressed from early development of the female reproductive tract from 12.5 dpc [24]–[26].

A complete assessment of preimplantation embryos showed that fertilization and embryo development were normal in the *Amhr2-cre;Stat3^fl/fl^* mice. The number of implantations was also not affected, but a significantly increased number of fetal resorptions demonstrates that the loss of stromal STAT3 altered the establishment of the feto-maternal interface. Given that inadequate placental development is a key underlying cause of fetal loss in midgestation [31] and in the absence of other overt defects in surviving fetuses, its seems reasonable to infer that disrupted placental development is the cause of demise of 50% of the implantation sites. The reason why some fetuses survive despite abnormal placentation while others do not is unclear, but similar incomplete penetrance is seen in other genetic models of placental development [31] including in mice deficient in the cytokine GM-CSF where a similarly expanded junctional zone is seen [32]. The significantly decreased fetal/placental weight ratio and histological evidence for thickened placental junctional zones are additional indicators of compromised placental function that are likely to originate earlier in placental morphogenesis and manifest as fetal demands accelerate in later gestation [31], [33].

The underlying cellular and molecular mechanisms for the effects of STAT3 deficiency on junctional zone development are not clear. There is substantial evidence that STAT3-regulating cytokines including LIF and IL6 have key roles as regulators of trophoblast differentiation and invasive function [34]–[36]. However, a direct role for STAT3 in trophoblasts is unlikely to explain the effects seen herein, as conceptus-derived tissues are hemizygous for STAT3 deficiency by virtue of their wild-type paternal genome, and STAT3 heterozygous embryos have no developmental defects. It is more likely that maternal tissues and particularly decidual cells arising from the uterine stroma are responsible for abnormal placental development, secondary to altered interactions with the maternal compartment of the implantation site. Notably, STAT3 responsiveness would be expected to be crucial for IL-11-regulated changes in decidual formation [37], and other STAT3 signalling agents have been implicated in related roles [38]. It is also possible that signals originating from immune cells play a part via STAT3 activation in uterine stromal cells to promote the progress of trophoblast implantation and development of the fully functional placenta.

Abundant STAT3, its phosphorylation and nuclear localisation in granulosa and cumulus cells throughout follicle development and ovulation [6] suggests it is involved in folliculogenesis. In the present study this could not be determined due to very inefficient Cre-mediated recombination of the floxed *Stat3* alleles in these cell types. The *Amhr2-cre* line has been successfully used for gene disruption in granulosa cells [39]–[41], including in our hands [42], but failed to mediate any change in granulosa STAT3 protein levels in follicles of any stage. This Cre expressing line used to generate granulosa-conditional *Dicer* mutation caused a follicle growth defect within 8 days postnatal indicating efficient expression from early stage follicle development [40]. The same line was also effective for showing a role for Dicer in uterine stroma [43]. Our finding that granulosa STAT3 could not be depleted by this experimental approach suggests to us that there may be a regulatory interaction between STAT3 and the Amh/Amhr2 pathway in granulosa cells as has been previously suggested [12].

STAT3 is also strongly and reproducibly detectable in oocyte cytoplasm from the primordial stage [6], [8], [44]. We achieved efficient deletion of STAT3 specifically in oocytes in the *Zp3-cre;Stat3^fl/fl^* genotype females. The *Zp3* gene is active from the early primary stage of follicle growth and our immunohistochemistry demonstrated reduced STAT3 abundance in oocytes of follicles at all growing stages. The oocyte is considered a likely target for a range of cytokines and growth factors that have the capacity to signal through the STAT3 pathway [9]. That the ablation of STAT3 did not affect fertility in terms of ovulation rate or live birth rate, indicates that oocyte STAT3 is not essential for oocyte development, maturation, fertilization or subsequent embryo development. Since we found no requirement for oocyte STAT3 we suggest that either the above mentioned growth factors play no major role directly in the developing oocyte, or their signalling in oocytes utilises another signal transduction pathway such as another STAT family member.

It is noteworthy that our results do not support a requirement for STAT3 within the early preimplantation embryo. Maternal transcripts for STAT3 transmitted from the oocyte are implicated in signalling mediated by both endogenous and maternal tract-derived cytokines LIF and IL-6 [16], [17]. Unlike in oocytes, the JAK/STAT pathway is constitutively activated in preimplantation mouse embryos with evidence of phosphorylated STAT3 in the nucleus throughout the preimplantation period [45]. Studies in *Lif* null mutant mice show that LIF acts from the 4 cell stage to phosphorylate STAT3, an important regulator of the OCT4-NANOG circuitry required to maintain embryonic stem cell self-renewal. IL6 and LIF have been implicated in activating STAT3-regulated anti-apoptotic pathways from early cleavage stages [46], [47]. However, it seems that although STAT3 signalling is essential for maintenance of pluripotent inner cell mass lineages, blastocyst formation can occur normally in the absence of STAT3 [46]. The current results are consistent with this latter finding.

In conclusion, this study is the first to find that STAT3 plays an important role specifically in uterine stromal cells during embryo implantation and the development of a functional placenta. The absence of STAT3 in this compartment caused a failure of gestation, dysmorphic placental development and in many conceptuses, embryo death and resorption. In oocytes STAT3 had no discernable role since recombination efficiently ablated the *Stat3* gene and resultant protein abundance, but had no effect on oocyte development or fertilization. Finally, the inability to delete STAT3 in granulosa cells using Cre-recombinase expression driven by the Amhr2 promoter suggests a regulatory link between STAT3 and Amhr2 such that the latter cannot be used to efficiently delete the former and hence no conclusion could be drawn on the role for Stat3 in granulosa cells.
